# Supplementary material for: Homeostatic microglia initially seed and activated microglia later reshape amyloid plaques in Alzheimer’s Disease
Source: Nat Commun. 2024 Dec 5;15:10634. doi: 10.1038/s41467-024-54779-w (PMC11621353; doi:10.1038/s41467-024-54779-w)
Supplement: Supplementary file 1 — Supplementary Information [file 41467_2024_54779_MOESM1_ESM.pdf]

Homeostatic microglia initially seed and activated microglia later reshape amyloid plaques in Alzheimer's Disease

Supplementary Information

Supplementary Figures

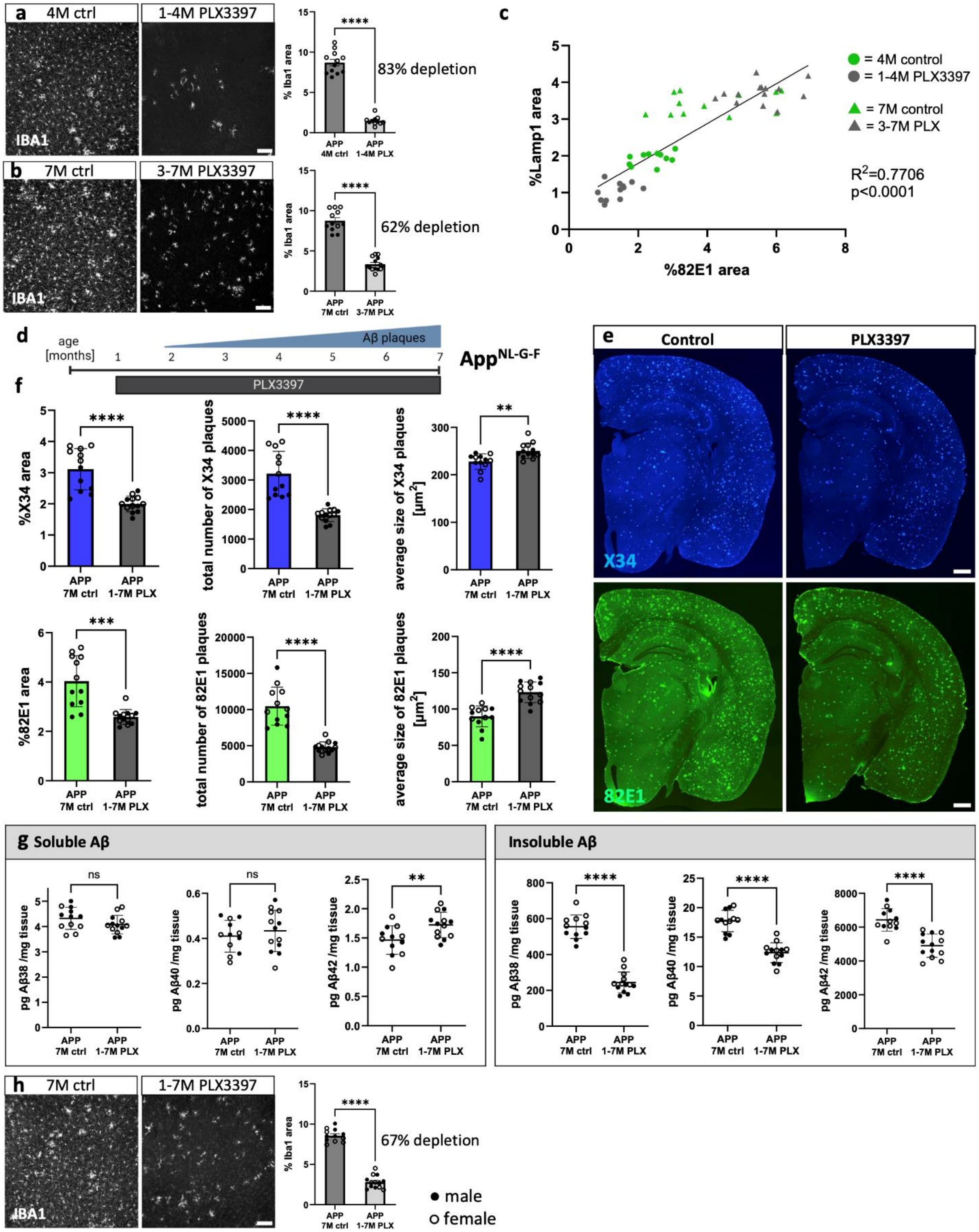

Supplementary Fig. 1: Additional information on PLX3397-treatment in *App*$^{NL\text{-}G\text{-}F}$ mice. (**a**) Representative images and microglia depletion efficiency of early PLX3397-treatment in *App*$^{NL\text{-}G\text{-}F}$ mice (APP) (two-tailed t-

test with Welch's correction, n(ctrl)=12, n(PLX)=11, p<0.0001). (**b**) Representative images and quantification of microglia depletion efficiency of late PLX3397-treatment in *App*$^{NL\text{-}G\text{-}F}$ mice (two-tailed t-test, n(ctrl)=12, n(PLX)=14, p<0.0001). (**c**) Linear regression of 82E1$^+$ plaque staining and LAMP1$^+$ dystrophic neurite staining across early and late microglia depletion cohorts (R^2=0.7706, p<0.0001). (**d**) Treatment scheme for sustained microglia depletion. *App*$^{NL\text{-}G\text{-}F}$ mice (APP) were fed PLX3397 from 1 month until 7 months of age (1-7M PLX) or control diet (7M ctrl). Created in BioRender. Cherretté, E. (2024) https://BioRender.com/u17o338. (**e**) Representative images of amyloid plaques in the brain, stained with X34 (fibrillar plaques) and 82E1 (total Aβ). (**f**) Image quantifications of X34$^+$ and 82E1$^+$ plaques in the whole brain (%X34 area: two-tailed t-test with Welch's correction, n(ctrl)=12, n(PLX)=13, p<0.0001; total number of X34 plaques: two-tailed t-test with Welch's correction, n(ctrl)=12, n(PLX)=13, p<0.0001; average size X34 plaques: two-tailed t-test, n(ctrl)=12, n(PLX)=13, p=0.0023; %82E1 area: two-tailed t-test with Welch's correction, n(ctrl)=12, n(PLX)=13, p=0.0005; total number of 82E1 plaques: two-tailed t-test with Welch's correction, n(ctrl)=12, n(PLX)=13, p<0.0001; average size 82E1 plaques: two-tailed t-test, n(ctrl)=12, n(PLX)=13, p<0.0001). (**g**) ELISA of amyloid levels in soluble and insoluble cortex extracts (sol. Aβ38: two-tailed t-test, n(ctrl)=12, n(PLX)=13, p=0.1392; sol. Aβ40: two-tailed t-test, n(ctrl)=12, n(PLX)=13, p=0.4911; sol. Aβ42: two-tailed t-test, n(ctrl)=12, n(PLX)=13, p=0.0098; insol. Aβ38: two-tailed t-test, n(ctrl)=12, n(PLX)=13, p<0.0001; insol. Aβ40: two-tailed t-test, n(ctrl)=12, n(PLX)=13, p<0.0001; insol. Aβ42: two-tailed t-test, n(ctrl)=12, n(PLX)=13, p<0.0001). (**h**) Representative images and quantification of microglia depletion efficiency after sustained PLX3397 treatment (two-tailed t-test, n(ctrl)=12, n(PLX)=13, p<0.0001). White dots represent female mice and black dots represent male mice. Scale bars 100 µm (a, b, h) and 500 µm (e). All data is presented as mean ± SD. *p≤0.05; **p≤0.01; ***p≤0.001; ****p≤0.0001. Source data are provided as a Source Data file.

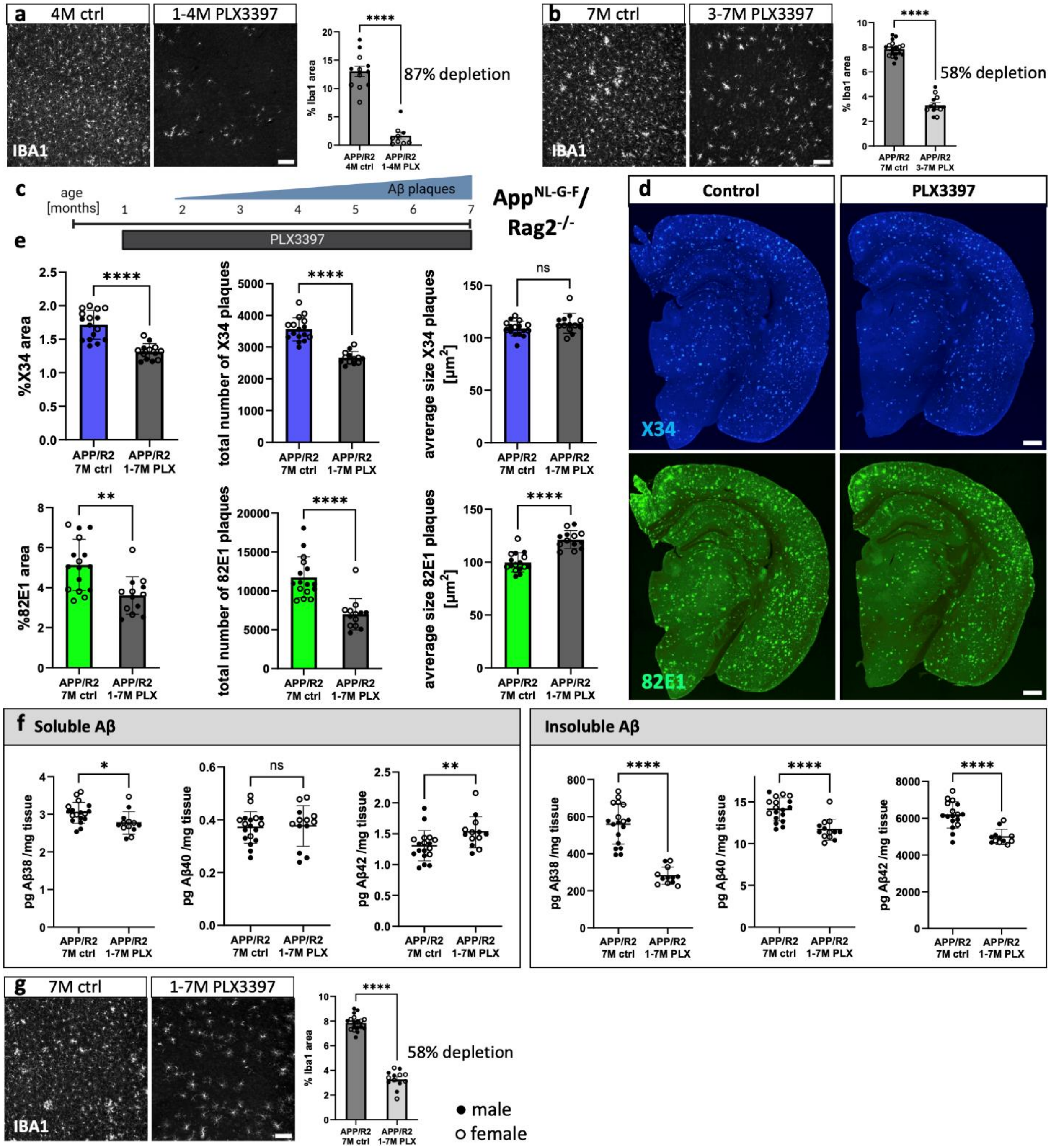

Supplementary Fig. 2: Additional information on PLX3397-treatment in *App^NL-G-F^/Rag2^-/-^* mice. **(a)** Microglia depletion efficiency of early PLX3397-treatment in *App^{NL-G-F}/Rag2$^{-/-}$* mice (APP/R2) (two-tailed t-test, n(ctrl)=12, n(PLX)=9, p<0.0001)**. (b)** Representative images and quantification of microglia depletion efficiency of late PLX3397-treatment in *App^{NL-G-F}/Rag2$^{-/-}$* mice (APP/R2) (two-tailed t-test, n(ctrl)=18, n(PLX)=13, p<0.0001). (**c**) Treatment scheme for sustained microglia depletion. *App^{NL-G-F}/Rag2$^{-/-}$* mice (APP/R2) were fed PLX3397 from 1 month until 7 months of age (1-7M PLX) or control diet (7M ctrl). Created in BioRender. Cherretté, E. (2024) https://BioRender.com/u17o338. (**d**) Representative images of amyloid plaques in the brain, stained with X34 (fibrillar plaques) and 82E1 (total Aβ). (**e**) Image quantifications of X34^{+} and 82E1^{+} plaques in the whole brain (%X34 area: two-tailed t-test, n(ctrl)=16,

n(PLX)=13, p<0.0001; total number of X34 plaques: two-tailed t-test with Welch's correction, n(ctrl)=16, n(PLX)=13, p<0.0001; average size X34 plaques: two-tailed t-test, n(ctrl)=16, n(PLX)=13, p=0.1188; %82E1 area: two-tailed t-test, n(ctrl)=16, n(PLX)=13, p=0.0013; total number of 82E1 plaques: two-tailed Mann-Whitney test, n(ctrl)=16, n(PLX)=13, p<0.0001; average size 82E1 plaques: two-tailed t-test, n(ctrl)=16, n(PLX)=13, p<0.0001). (**f**) ELISA of amyloid levels in soluble and insoluble cortex extracts (sol. Aβ38: two-tailed t-test, n(ctrl)=18, n(PLX)=13, p=0.0146; sol. Aβ40: two-tailed t-test, n(ctrl)=18, n(PLX)=13, p=0.8164; sol. Aβ42: two-tailed Mann-Whitney test, n(ctrl)=18, n(PLX)=13, p=0.0073; insol. Aβ38: two-tailed t-test with Welch's correction, n(ctrl)=18, n(PLX)=12, p<0.0001; insol. Aβ40: two-tailed t-test, n(ctrl)=18, n(PLX)=13, p<0.0001; insol. Aβ42: two-tailed t-test, n(ctrl)=17, n(PLX)=12, p<0.0001). (**g**) Representative images and quantification of microglia depletion efficiency after sustained PLX3397 treatment (two-tailed t-test, n(ctrl)=18, n(PLX)=13, p<0.0001). White dots represent female mice and black dots represent male mice. Scale bars 100 µm (a, b, g) and 500 µm (d). All data is presented as mean ± SD. *p≤0.05; **p≤0.01; ***p≤0.001; ****p≤0.0001. Source data are provided as a Source Data file.

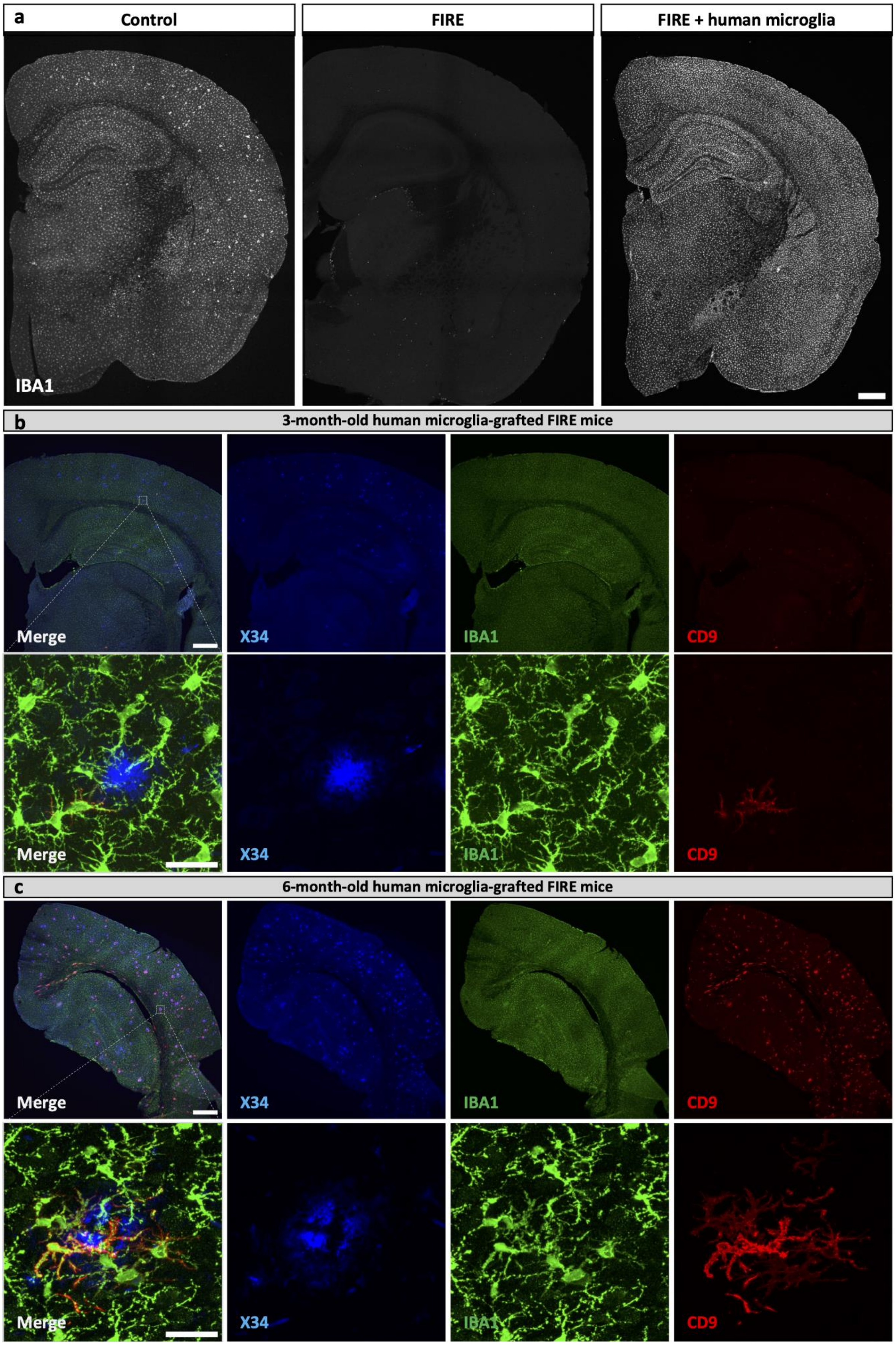

a
Control
FIRE
FIRE + human microglia
IBA1
b
3-month-old human microglia-grafted FIRE mice
Merge
X34
IBA1
CD9
Merge
X34
IBA1
CD9
c
6-month-old human microglia-grafted FIRE mice
Merge
X34
IBA1
CD9
Merge
X34
IBA1
CD9

Supplementary Fig. 3: Human microglia grafted into FIRE mice populate the whole brain and upregulate the DAM marker CD9 around amyloid plaques. (**a**) Images of microglia in the brain of control mice (*App*$^{NL-G-F}$*; Rag2*$^{-/-}$*; IL2rg*$^{-/-}$*; hCSF1*^{KI}, with mouse microglia), FIRE mice (*App*$^{NL-G-F}$*; Rag2*$^{-/-}$*; IL2rg*$^{-/-}$*; hCSF1*^{KI}*; Csf1r*$^{\Delta FIRE/\Delta FIRE}$, no microglia), and FIRE mice xenotransplanted with human microglia. (**b-c**) Overview and higher magnification images of human grafted microglia at 3 months (**b**) and 6 months (**c**) around amyloid plaques stained with IBA1 and the DAM marker CD9. Scale bars 500 µm (a,b,c) and 30 µm (b,c lower panel).

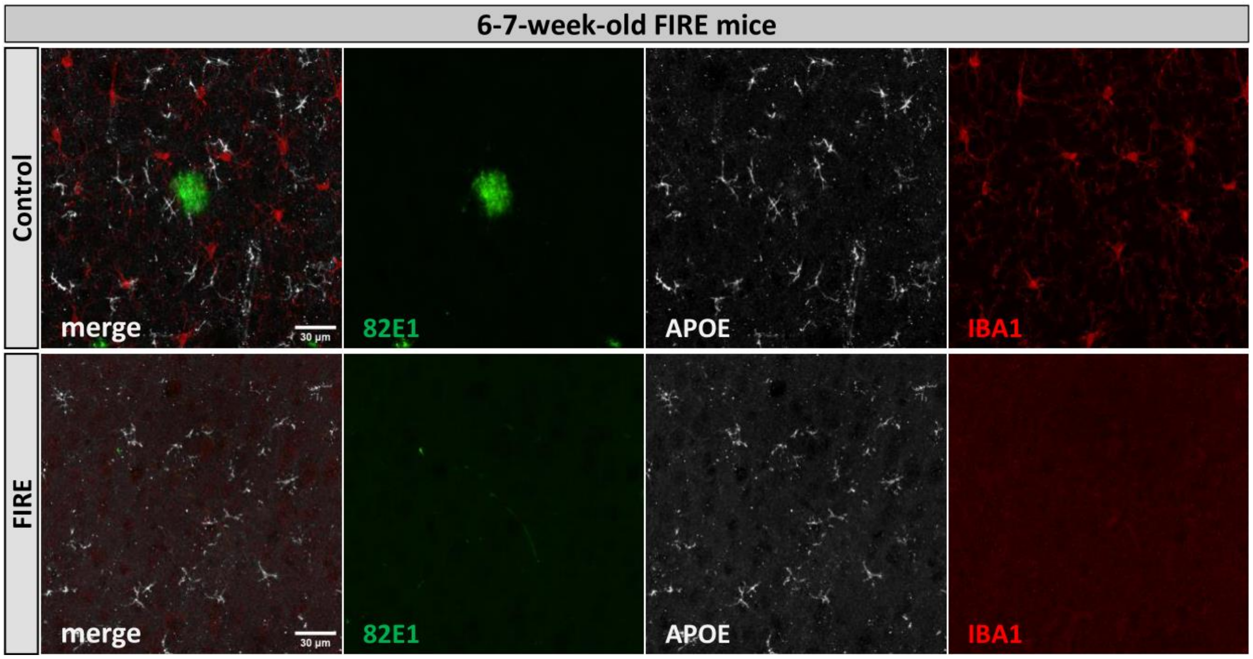

Supplementary Fig. 4: In 6-7-week-old control and FIRE mice, APOE expression is driven by non-microglial cells. Representative images of amyloid plaques, APOE, and microglia in the brain of 6-7-week-old control mice (*App*$^{NL\text{-}G\text{-}F}$*; Rag2*$^{-/-}$*; IL2rg*$^{-/-}$*; hCSF1*^{KI}*,* with mouse microglia) and FIRE mice (*App*$^{NL\text{-}G\text{-}F}$*; Rag2*$^{-/-}$*; IL2rg*$^{-/-}$*; hCSF1*^{KI}*; Csf1r*$^{\Delta FIRE/\Delta FIRE}$, no microglia). Scale bars 30µm.

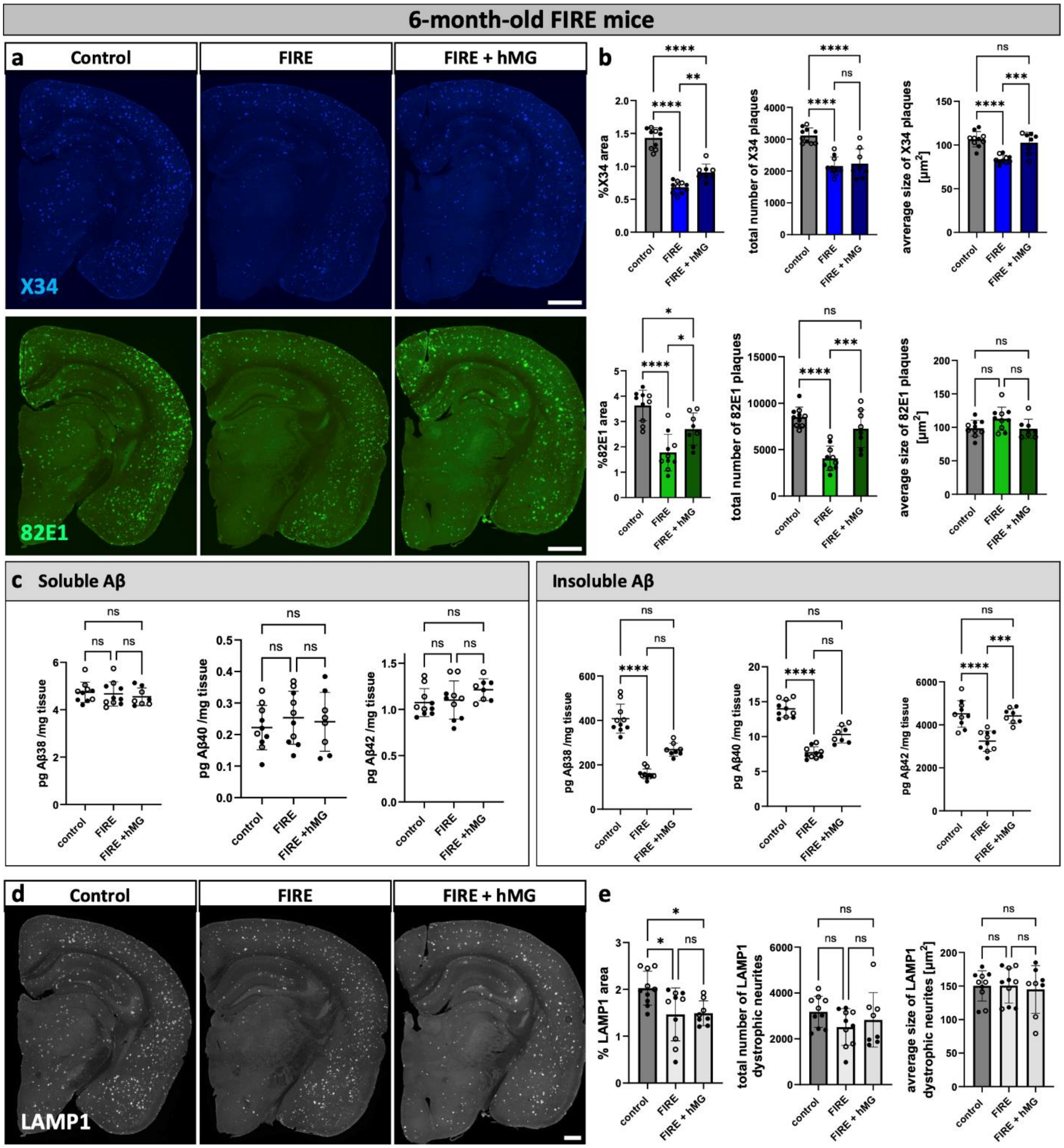

Supplementary Fig. 5: Reduced plaque burden in 6-month-old FIRE mice and rescue by human microglia transplantation. (**a**) Images of X34⁺ and 82E1⁺ amyloid plaques in the whole brain of control mice (*$App^{NL\text{-}G\text{-}F}$; $Rag2^{-/-}$; $IL2rg^{-/-}$; $hCSF1^{KI}$*, with mouse microglia), FIRE mice (*$App^{NL\text{-}G\text{-}F}$; $Rag2^{-/-}$; $IL2rg^{-/-}$; $hCSF1^{KI}$; $Csf1r^{\Delta FIRE/\Delta FIRE}$*, no microglia), and xenografted FIRE mice (FIRE + hMG, human microglia). (**b**) Image quantifications of amyloid plaques in the whole brain (%X34 area: one-way ANOVA, n(ctrl)=10, n(FIRE)=10, n(FIRE +hMG)=8, p<0.0001; total number of X34 plaques: one-way ANOVA, n(ctrl)=10, n(FIRE)=10, n(FIRE +hMG)=8, p<0.0001; average size X34 plaques: one-way ANOVA, n(ctrl)=10, n(FIRE)=10, n(FIRE +hMG)=8, p<0.0001; %82E1 area: one-way ANOVA, n(ctrl)=10, n(FIRE)=10, n(FIRE +hMG)=8, p<0.0001; total number of 82E1 plaques: one-way ANOVA, n(ctrl)=10, n(FIRE)=10, n(FIRE +hMG)=8, p<0.0001; average size 82E1 plaques: one-way ANOVA, n(ctrl)=10, n(FIRE)=10, n(FIRE +hMG)=8, p=0.0594). (**c**) ELISA of soluble and

insoluble Aβ levels in the cortex (sol. Aβ38: one-way Kruskal-Wallis test, n(ctrl)=10, n(FIRE)=10, n(FIRE +hMG)=8, p=0.6434; sol. Aβ40: one-way ANOVA, n(ctrl)=10, n(FIRE)=10, n(FIRE +hMG)=8, p=0.6971; sol. Aβ42: one-way ANOVA, n(ctrl)=10, n(FIRE)=10, n(FIRE +hMG)=8, p=0.2007; insol. Aβ38: one-way Kruskal-Wallis test, n(ctrl)=10, n(FIRE)=10, n(FIRE +hMG)=8, p<0.0001; insol. Aβ40: one-way Kruskal-Wallis test, n(ctrl)=10, n(FIRE)=10, n(FIRE +hMG)=8, p<0.0001; insol. Aβ42: one-way ANOVA, n(ctrl)=10, n(FIRE)=10, n(FIRE +hMG)=8, p<0.0001). (**d**) Images of $LAMP1^+$ dystrophic neurites in the whole brain. (**e**) Image quantifications $LAMP1^+$ dystrophic neurites in the whole brain (%LAMP1 area: one-way ANOVA, n(ctrl)=10, n(FIRE)=10, n(FIRE +hMG)=8, p=0.0118; total number of LAMP1 dystrophic neurites: one-way ANOVA, n(ctrl)=10, n(FIRE)=10, n(FIRE +hMG)=8, p=0.2678; average size of LAMP1 dystrophic neurites: one-way Kruskal-Wallis test, n(ctrl)=10, n(FIRE)=10, n(FIRE +hMG)=8, p=0.8563). White dots represent female mice and black dots represent male mice. Scale bars 500 µm (a,d). All data is presented as mean ± SD. *p≤0.05; **p≤0.01; ***p≤0.001; ****p≤0.0001. Source data are provided as a Source Data file.

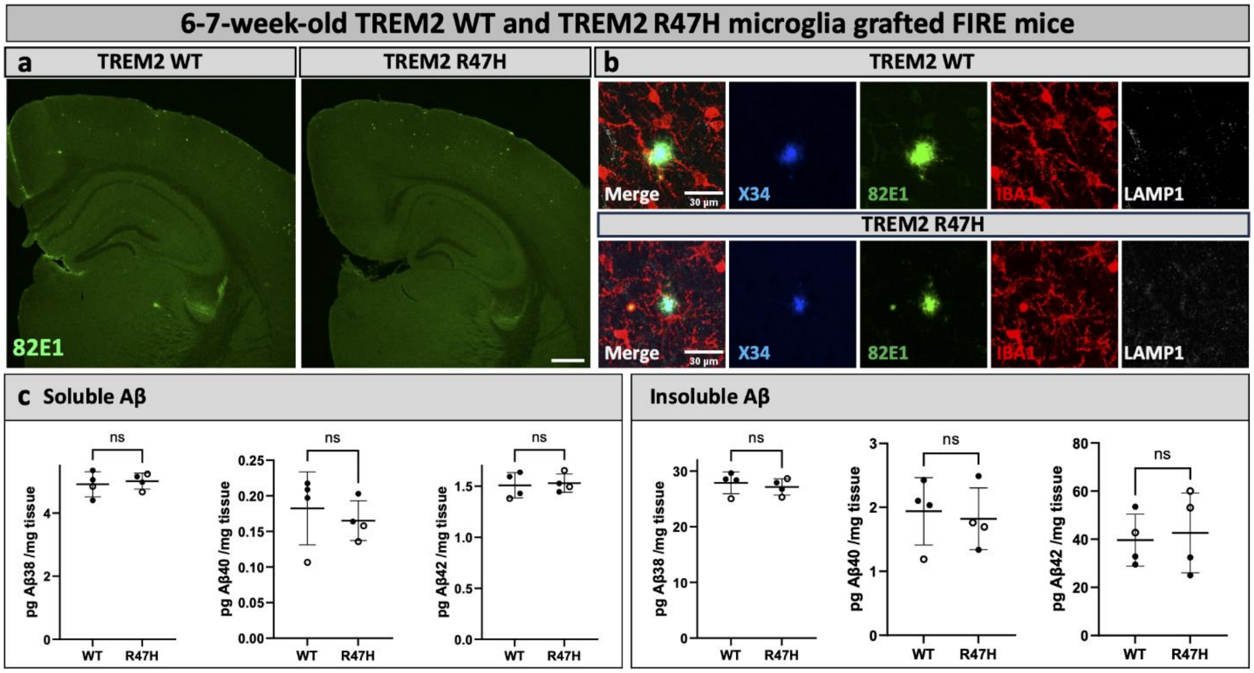

Supplementary Fig. 6: Amyloid pathology in 6-7-week-old FIRE mice transplanted with wildtype and *TREM2*$^{R47H/R47H}$ human microglia. FIRE mice (*App*$^{NL\text{-}G\text{-}F}$; *Rag2*$^{-/-}$; *IL2rg*$^{-/-}$; *hCSF1*^{KI}; *Csf1r*$^{\Delta FIRE/\Delta FIRE}$) were xenotransplanted with human WT microglia (WT) and human microglia harboring the *Trem2*$^{R47H/R47H}$ (R47H) risk gene at P4 and analyzed at 6-7 weeks of age. (**a**) Overview images of amyloid plaques in the cortex of WT and R47H microglia grafted mice. (**b**) Higher magnification images on amyloid plaques, surrounded by human microglia. Early amyloid plaques do not yet show LAMP1^{+} dystrophic neurites. (**c**) ELISA of soluble and insoluble Aβ extracts of the cortex (sol. Aβ38: two-tailed t-test, n(WT)=4, n(R47H)=13, p=0.6948; sol. Aβ40: two-tailed t-test, n(WT)=4, n(R47H)=13, p=0.5737; sol. Aβ42: two-tailed t-test, n(WT)=4, n(R47H)=13, p=0.7904; insol. Aβ38: two-tailed t-test, n(WT)=4, n(R47H)=13, p=0.5686; insol. Aβ40: two-tailed t-test, n(WT)=4, n(R47H)=13, p=0.7534; insol. Aβ42: two-tailed t-test, n(WT)=4, n(R47H)=13, p=0.7714). White dots represent female mice and black dots represent male mice. Scale bars 500 µm (a) and 30µm (b). All data is presented as mean ± SD. Source data are provided as a Source Data file.

Supplementary Table

Study	Mouse model	Treatment	Depletion efficiency	Effect on plaque load	Explained
APP/PS1 (plaque deposition from ~1.5 months)					
Grathwohl et al., 2009[1]	*APP/PS1; CD11b-HSVTK*	6-9 weeks or 5-6M GCV	90-95%	**Histology (6-9 weeks):** no change in Congo Red amyloid plaques<br>**Aβ Western Blot (5-6M)**: no change<br>**Histology (5-6M):** no change in Congo Red amyloid plaques	yes
Olmos-Alonso et al., 2016[2]	*APP/PS1*	6-9M GW2580	30%	**Soluble Aβ ELISA**: no change<br>**Histology:** no change in number of 6E10[+] amyloid plaques in cortex	yes
Unger et al., 2018[3]	*APP/PS1*	12-13M PLX5622	70-90%	**Histology**: no change in number or mean area of Thioflavin+ plaques in hippocampus and cortex	yes
Zhao et al., 2017[4]	*Cx3cr1$^{CreER/+}$:R26$^{DTR/+}$; APP/PS1*	Diphtheria toxin at 15M,	99%, repopulation in 2nd week	**Intravital time-lapse imaging:** no changes in the number of Congo Red[+] Aβ plaques,<br>but an increase in size	yes
5xFAD (plaque deposition from ~2 months)					
Spangenberg et al., 2019[5]	*5xFAD*	1.5- 4M or 1.5-7M PLX5622, repopulation	97–100%	**Aβ ELISA:** no changes in soluble or insoluble Aβ or Aβ42<br>**Histology:** reduced Thio-S[+] and 6E10[+] plaque number and volume<br>Increased CAA in Cortex and thalamus	yes
Delizannis et al., 2021[6]	*5xFAD with AD-Tau injection*	1.5M-6M PLX3397	92%	**Histology:** reduced NAB228[+] Aβ signal in the cortex (layers 4-6), but not subiculum<br>Reduced plaque size in layers 4-6 of the cortex	yes
Sosna et al., 2018[7]	*5xFAD*	2-5M PLX3397	99%	**Histology:** reduction of 6E10[+] amyloid area and size	yes
Shabestari et al., 2022[8]	*5xFAD; FIRE*	0 – 5M or 0-6M	100%	**Aβ ELISA:** reduced insoluble Aβ40 and 42, no change in soluble Aβ<br>**Histology:** reduced parenchymal plaques (AmyloGlo) in cortex, hippocampus, and Thalamus, increased CAA,	yes
Casali et al., 2020 [9]	*5xFAD*	4-5M PLX5622, repopulation	50%	**Histology:** Slightly reduced 6E10 area in the thalamus, not in other brain regions<br>increased diffuse-like plaques and fewer compact-like plaques	partially
Son et al., 2020[10]	*5xFAD*	9-10M PLX3397	50%	**Histology:** reduced 6E10 Aβ deposition<br>**Western Blot:** decreased APP levels CTFβ, and Aβ in the cortex	no
Spangenberg et al., 2016[11]	*5xFAD*	10-11M PLX3397	90%	**Aβ ELISA:** no change in soluble or insoluble Aβ levels<br>**Histology:** no change in Thio-S[+] and 6E10[+] plaque numbers or size	yes
App$^{NL\text{-}G\text{-}F}$ (plaque deposition from ~1.5 months)					
Benitez et al., 2021[12]	*App$^{NL\text{-}G\text{-}F}$*	1.5-3.5M PLX5622	nearly all microglia depleted	**Histology:** reduction in LCO[+] plaque density of small plaques (hippocampus),	yes
Clayton et al., 2021[13]	*App$^{NL\text{-}G\text{-}F}$ (with AAV-P301L-tau injection)*	4M-6M PLX5622	nearly all microglia depleted	**Histology:** increased Thio-S area, number of plaques, and average size of plaques<br>Increased 4G8 area and plaque size	yes

Supplementary Table 1: Overview of microglia depletion studies in amyloid mice. This table summarizes the studies investigating the effect of microglia depletion on amyloid plaques. References, mouse model used, type of treatment and timing, microglia depletion efficiency, effect on amyloid plaques, and whether or not the results can be explained with our findings are indicated. Gray highlights indicate minor details that do not align with our observations.

Supplementary References

1. Grathwohl, S. A. *et al.* Formation and maintenance of Alzheimer's disease β-amyloid plaques in the absence of microglia. *Nat Neurosci* 12, 1361–1363 (2009).

2. Olmos-Alonso, A. *et al.* Pharmacological targeting of CSF1R inhibits microglial proliferation and prevents the progression of Alzheimer's-like pathology. *Brain* 139, 891–907 (2016).

3. Unger, M. S., Schernthaner, P., Marschallinger, J., Mrowetz, H. & Aigner, L. Microglia prevent peripheral immune cell invasion and promote an anti-inflammatory environment in the brain of APP-PS1 transgenic mice. *J Neuroinflammation* 15, (2018).

4. Zhao, R., Hu, W., Tsai, J., Li, W. & Gan, W. B. Microglia limit the expansion of β-amyloid plaques in a mouse model of Alzheimer's disease. *Mol Neurodegener* 12, (2017).

5. Spangenberg, E. *et al.* Sustained microglial depletion with CSF1R inhibitor impairs parenchymal plaque development in an Alzheimer's disease model. *Nat Commun* 10, 1–21 (2019).

6. Delizannis, A. T. *et al.* Effects of microglial depletion and TREM2 deficiency on Aβ plaque burden and neuritic plaque tau pathology in 5XFAD mice. *Acta Neuropathol Commun* 9, (2021).

7. Sosna, J. *et al.* Early long-term administration of the CSF1R inhibitor PLX3397 ablates microglia and reduces accumulation of intraneuronal amyloid, neuritic plaque deposition and pre-fibrillar oligomers in 5XFAD mouse model of Alzheimer's disease. *Mol Neurodegener* 13, 1–11 (2018).

8. Kiani Shabestari, S. *et al.* Absence of microglia promotes diverse pathologies and early lethality in Alzheimer's disease mice. *Cell Rep* 39, (2022).

9. Casali, B. T., MacPherson, K. P., Reed-Geaghan, E. G. & Landreth, G. E. Microglia depletion rapidly and reversibly alters amyloid pathology by modification of plaque compaction and morphologies. *Neurobiol Dis* 142, 104956 (2020).

10. Son, Y. *et al.* Inhibition of colony-stimulating factor 1 receptor by plx3397 prevents amyloid beta pathology and rescues dopaminergic signaling in aging 5xfad mice. *Int J Mol Sci* 21, 1–14 (2020).

11. Spangenberg, E. E. *et al.* Eliminating microglia in Alzheimer's mice prevents neuronal loss without modulating amyloid-β pathology. *Brain* 139, 1265–1281 (2016).

12. Benitez, D. P. *et al.* Knock-in models related to Alzheimer's disease: synaptic transmission, plaques and the role of microglia. *Mol Neurodegener* 16, (2021).

13. Clayton, K. *et al.* Plaque associated microglia hyper-secrete extracellular vesicles and accelerate tau propagation in a humanized APP mouse model. *Mol Neurodegener* 16, (2021).
