# Supplementary material for: Homeostatic microglia initially seed and activated microglia later reshape amyloid plaques in Alzheimer’s Disease
Source: Nat Commun. 2024 Dec 5;15:10634. doi: 10.1038/s41467-024-54779-w (PMC11621353; doi:10.1038/s41467-024-54779-w)
Supplement: Supplementary file 2 — Reporting Summary [file 41467_2024_54779_MOESM2_ESM.pdf]

nature portfolio

Corresponding author(s): Bart De Strooper

Last updated by author(s): Nov 13, 2024

Reporting Summary

Nature Portfolio wishes to improve the reproducibility of the work that we publish. This form provides structure for consistency and transparency in reporting. For further information on Nature Portfolio policies, see our Editorial Policies and the Editorial Policy Checklist.

Statistics

For all statistical analyses, confirm that the following items are present in the figure legend, table legend, main text, or Methods section.

n/a	Confirmed	
☐	☒	The exact sample size (*n*) for each experimental group/condition, given as a discrete number and unit of measurement
☐	☒	A statement on whether measurements were taken from distinct samples or whether the same sample was measured repeatedly
☐	☒	The statistical test(s) used AND whether they are one- or two-sided<br>*Only common tests should be described solely by name; describe more complex techniques in the Methods section.*
☒	☐	A description of all covariates tested
☐	☒	A description of any assumptions or corrections, such as tests of normality and adjustment for multiple comparisons
☐	☒	A full description of the statistical parameters including central tendency (e.g. means) or other basic estimates (e.g. regression coefficient) AND variation (e.g. standard deviation) or associated estimates of uncertainty (e.g. confidence intervals)
☐	☒	For null hypothesis testing, the test statistic (e.g. *F*, *t*, *r*) with confidence intervals, effect sizes, degrees of freedom and *P* value noted<br>*Give P values as exact values whenever suitable.*
☒	☐	For Bayesian analysis, information on the choice of priors and Markov chain Monte Carlo settings
☒	☐	For hierarchical and complex designs, identification of the appropriate level for tests and full reporting of outcomes
☒	☐	Estimates of effect sizes (e.g. Cohen's *d*, Pearson's *r*), indicating how they were calculated

Our web collection on statistics for biologists contains articles on many of the points above.

Software and code

Policy information about availability of computer code

Data collection	No code was used for data collection in this study.
Data analysis	Image quantifications of confocal images were perfomed with Fiji (Image J) software.<br>Statistical analysis was performed with GraphPad Prism 10.

For manuscripts utilizing custom algorithms or software that are central to the research but not yet described in published literature, software must be made available to editors and reviewers. We strongly encourage code deposition in a community repository (e.g. GitHub). See the Nature Portfolio guidelines for submitting code & software for further information.

Data

Policy information about availability of data

All manuscripts must include a data availability statement. This statement should provide the following information, where applicable:

- Accession codes, unique identifiers, or web links for publicly available datasets
- A description of any restrictions on data availability
- For clinical datasets or third party data, please ensure that the statement adheres to our policy

All data necessary for the conclusions of the study are available in the main text, figures and supplementary figures. Source data are provided as a Source Data file.

Research involving human participants, their data, or biological material

Policy information about studies with human participants or human data. See also policy information about sex, gender (identity/presentation), and sexual orientation and race, ethnicity and racism.

Reporting on sex and gender	No human participants were used in this study.
Reporting on race, ethnicity, or other socially relevant groupings	Not applicable.
Population characteristics	Not applicable.
Recruitment	Not applicable.
Ethics oversight	Not applicable.

Note that full information on the approval of the study protocol must also be provided in the manuscript.

Field-specific reporting

Please select the one below that is the best fit for your research. If you are not sure, read the appropriate sections before making your selection.

☒ Life sciences ☐ Behavioural & social sciences ☐ Ecological, evolutionary & environmental sciences

For a reference copy of the document with all sections, see nature.com/documents/nr-reporting-summary-flat.pdf

Life sciences study design

All studies must disclose on these points even when the disclosure is negative.

Sample size	Sample size was estimated based on previous experiments performed in the lab. To calculate the number of mice needed for IF and MSD ELISA, sample size we conducted a priori power analyses using G*Power (sample size noted throughout the test). The expected effect size was based on previous publications or, when possible, data previously generated in the lab. Alpha level and power were set at 5% and 80%, respectively, and the statistical model used for the sample size calculation was a t-test.
Data exclusions	A few data points were excluded from the study due to damaged samples or technical errors. All excluded data point and reasons for exclusion is indicated in the source data files.
Replication	Experiments have been performed independently. We reproduced the same findings in independent experiments and different mouse strains (immunocompetent and immunodeficient), and with pharmacological and genetic manipulation. Our data can explain previous findings in the literature.
Randomization	Animals were randomly assigned to conditions and conditions to account for potential ordering effects. To avoid litter bias in the mouse experiments, experimental groups were composed of animals from different litters randomly distributed. Both sexes were used at similar ratios.
Blinding	The investigators were not blinded during data collection and analysis. Blinding was not necessary because image analysis was performed semi-automated or fully automated (when possible).

Reporting for specific materials, systems and methods

We require information from authors about some types of materials, experimental systems and methods used in many studies. Here, indicate whether each material, system or method listed is relevant to your study. If you are not sure if a list item applies to your research, read the appropriate section before selecting a response.

Materials & experimental systems

n/a	Involved in the study	
☐	☒	Antibodies
☐	☒	Eukaryotic cell lines
☒	☐	Palaeontology and archaeology
☐	☒	Animals and other organisms
☒	☐	Clinical data
☒	☐	Dual use research of concern
☒	☐	Plants

Methods

n/a	Involved in the study	
☒	☐	ChIP-seq
☒	☐	Flow cytometry
☒	☐	MRI-based neuroimaging

Antibodies

Antibodies used	Mouse anti-Human Amyloidβ (N) (82E1) (1/200, IBL, Cat#10323, clone 820)<br>Rabbit anti-Iba1 (1/200, WAKO, Cat#019-19741)<br>Guinea pig anti-Iba1 (1/500, Synaptic Systems, Cat#234 308, clone Gp311H9)<br>Rat anti-Lamp1 (1/500, Santa Cruz, Cat#sc-19992, 1D4B)<br>Rabbit anti-ubiquitin (1/250, Abcam, Cat#ab134953, clone EPR8830)<br>Mouse anti-human CD9 (1/500, BioLegend, Cat#312102, clone HI9a)<br>Rabbit anti-human P2RY12 (1/500, Atlas antibodies, Cat#HPA014518)<br>Goat anti-APOE (1/1000, Chemicon International, Cat#AB947)<br>Donkey anti-mouse Alexa Fluor 488 (1/500, Invitrogen, Cat#A21202)<br>Donkey anti-rabbit Alexa Fluor 594 (1/500, Invitrogen, Cat#A21207)<br>Donkey anti-rat Alexa Fluor 647 (1/500, Abcam, Cat#ab150155)<br>Donkey anti-rabbit Alexa Fluor 488 (1/500, Invitrogen, Cat#A21206)<br>Donkey anti-mouse Alexa Fluor 594 (1/500, Invitrogen, Cat#A21203)<br>Donkey anti-guinea pig Cy5 (1/500, Jackson Immunolabs, Cat#706-175-148)<br>Donkey anti-goat Alexa Fluor 647 (1/500, Invitrogen, Cat#A21447)<br>LTDA-38 (Aβ ELISA capture antibodies generated in the lab)<br>LTDA-40 (Aβ ELISA capture antibodies generated in the lab)<br>LTDA-42 (Aβ ELISA capture antibodies generated in the lab)<br>LTDA-hAβN (Aβ ELISA detection antibodies generated in the lab)
Validation	Mouse anti-Human Amyloidβ (N) (82E1): validated in Horikoshi Y, et al. Development of Abeta terminal end-specific antibodies and sensitive ELISA for Abeta variant. Biochem Biophys Res Commun. 2004 Jul 2;319(3):733-7<br>Rabbit anti-Iba1: Validated by the company by IHC on mouse brain sections..<br>Guinea pig anti-Iba1: Validated by the company by IHC on rat, mouse and human brain sections.<br>Rat anti-Lamp1 : Validated by IHC by McNiven Laboratory, Mayo Clinic, Rochester, on AML12 mouse hepatocyte cells. Hughes EN, August JT. Characterization of plasma membrane proteins identified by monoclonal antibodies. J Biol Chem. 1981 Jan 25;256(2):664-71.<br>Rabbit anti-Ubiquitin : Validated in mouse liver tissue.<br>Mouse anti-human CD9: Validated on the BT474 breast cancer cell line.<br>Rabbit anti-human P2RY12: Validated by the company by IHC on sections of human cerebral cortex and liver tissue.<br>Donkey anti-APOE: Validated by several papers listed by the company.<br>LTDA-hAβN; Recombinant mouse monoclonal antibody with epitope at first 7 residues of human Aβ; requires β-cleavage of APP to recognize human APP-β-CTF and Aβ 1-X. Antibody is tested for human Aβ (western blot and ELISA) and β-CTF(western blot), control rodent Aβ and β-CTF are not recognized.<br>LTDA-38; Recombinant mouse monoclonal antibody recognizes the carboxy terminus of Aβ38, doesn't recognize Aβ37, Aβ40 or Aβ42 (ELISA). It reacts equally potent to rodent Aβ38.<br>LTDA-40; Recombinant mouse monoclonal antibody recognizes the carboxy terminus of Aβ40, doesn't recognize Aβ37, Aβ38 nor Aβ42 (ELISA). It reacts equally potent to rodent Aβ40.<br>LTDA-42; Recombinant mouse monoclonal antibody recognizes the carboxy terminus of Aβ42, doesn't recognize Aβ37, Aβ38 nor Aβ40 (ELISA). It reacts equally potent to rodent Aβ42

Eukaryotic cell lines

Policy information about cell lines and Sex and Gender in Research

Cell line source(s)	H9 (WA09)/ WiCell Research Institute / RRID:CVCL_9773, female<br>H9-TREM2R47H / TREM2R47H / KUL Stem Cell Institute / Claes et al., 2019
Authentication	Cell lines were authenticated by the providers by Karyotyping and whole genome sequencing, and have been tested for pluripotency (https://hpscreg.eu/cell-line/WAe009-A). The H9-TREM2R47H linewas tested for chromosomal alterations.
Mycoplasma contamination	All cell lines tested negative at least 3 times for Mycoplasma contamination.
Commonly misidentified lines<br>(See ICLAC register)	None of the cell lines used in this study is known to be cross-contaminated or otherwise misidentified, and is not listed in the Register of Misidentified Cell Lines from ICLAC.

Animals and other research organisms

Policy information about studies involving animals; ARRIVE guidelines recommended for reporting animal research, and Sex and Gender in Research

Laboratory animals	Mouse, Apptm3.1Tcs, 4-7 months of age<br>Mouse; Apptm3.1Tcs;Rag2tm1.1Cgn, 4-7 months of age<br>Mouse; Rag2tm1.1Flv; Csf1tm1(CSF1)Flv; Il2rgtm1.1Flv;Apptm3.1Tcs, 6 weeks-6 months of age<br>Mouse; Rag2tm1.1Flv; Csf1tm1(CSF1)Flv; Il2rgtm1.1Flv; Apptm3.1Tcs; Csf1Rem1Bdes, 6 weeks-6 months of age
Wild animals	No wild animals were used

Reporting on sex	Experimental groups were balanced in terms of the sex of the mice.<br>Data points from male and female mice are indicated in the graphs with black and white dots respectively.
Field-collected samples	No field-collected samples were used in this study.
Ethics oversight	Animal experiments were approved by the local Ethical Committee of Laboratory Animals of the KU Leuven (government licence LA1210579 and project P125/2022) following local and EU guideline.

Note that full information on the approval of the study protocol must also be provided in the manuscript.

Plants

Seed stocks	Not applicable.
Novel plant genotypes	Not applicable.
Authentication	Not applicable.
